# Adaptive and Maladaptive Networks Using Ecological Momentary Assessment

**DOI:** 10.32872/cpe.17891

**Published:** 2026-05-29

**Authors:** Bettina Hufschmidt, Mareike Ebert, Arwin Nemani, Viktoria Kohl, Lucie Pahlen, Simon Müller, Stefan G. Hofmann, Ulrich Stangier

**Affiliations:** 1 Department of Clinical Psychology and Psychotherapy, Goethe University Frankfurt, Frankfurt am Main, Germany; 2 Department of Psychology, Philipps-University of Marburg, Marburg, Germany; Friedrich-Alexander-Universität Erlangen-Nürnberg, Erlangen, Germany

**Keywords:** ecological momentary assessment, case conceptualization, maladaptive psychological processes, adaptive psychological processes, network approach

## Abstract

**Background:**

A person’s ability to adapt to a given context is a critical determinant of mental health and psychopathology, which has been redefined by network approaches and Ecological Momentary Assessment (EMA). This study examined whether including adaptive processes in EMA enhances the informational value of idiographic network models.

**Method:**

Forty-five university students participated in a multi-week EMA protocol assessing psychological dimensions using bipolar visual analogue scales ranging from maladaptive to adaptive. Participants were randomly assigned to two groups: one assessed only maladaptive processes; the other included both maladaptive and adaptive processes.

**Results:**

Network analyses indicated higher density and connectivity in the adaptive–maladaptive group, as well as significantly reduced floor effects across all variables. Greater response dispersion was associated with more differentiated network structures. Motivation emerged as the most central node across conditions, highlighting its relevance as a transdiagnostic treatment target. Cognitive processing showed strong associations with other variables, underlining its clinical importance.

**Conclusion:**

The findings suggest that incorporating adaptive dimensions into EMA facilitates a more comprehensive understanding of psychological functioning and improves the interpretability of idiographic models. The study represents an initial feasibility investigation and a basis for further investigations in clinical practice.

## Highlights

- Adaptive processes enrich EMA data by increasing response variability and network connectivity.
- Adaptive processes enhance EMA data, reducing floor effects and revealing psychological dynamics.
- Assessing motivational processes appears beneficial for enhancing psychological functioning.
- Targeting cognitive processes in EMA seems effective in promoting adaptive functioning.

Case conceptualization plays a crucial role in treatment planning, since it offers a context-sensitive, yet concise model of the client’s functioning and helps to identify relevant treatment targets along with appropriate assessment methods (Gilboa-Schechtman, 2024). Usually, retrospective reports are used as the primary source of information. However, they may be affected by a number of biases, e.g., overestimation of frequency and intensity of symptoms (Van den Bergh & Walentynowicz, 2016). Ecological Momentary Assessments (EMA) offer a potential solution to these limitations of retrospective self-reports. By collecting data in real time in people's everyday lives, EMA enables the capture of detailed information about the dynamics of psychological processes, including emotions, cognitions, and behaviors (Shiffman et al., 2008).

Recent advances in dynamic network analysis have provided a new perspective on using EMA data for treatment planning (Hayes et al., 2019). An increasing number of studies have provided evidence that dynamic network analyses may significantly enhance treatment planning by creating an idiographic dynamic model of the patient’s problem based on EMA data (Burger et al., 2020; Frumkin et al., 2021; Roefs et al., 2022; Rubel et al., 2018).

However, it is not yet clear how to select variables to provide a valid idiographic conceptualization of the patient’s problem. Many studies use symptom items based on diagnostic criteria or standardized psychometric instruments for EMA assessment (Cusack et al., 2024), thereby adopting a nomothetic approach, which may not be appropriate for the individual processes of the patient (Beltz et al., 2016).

**Figure 1:**
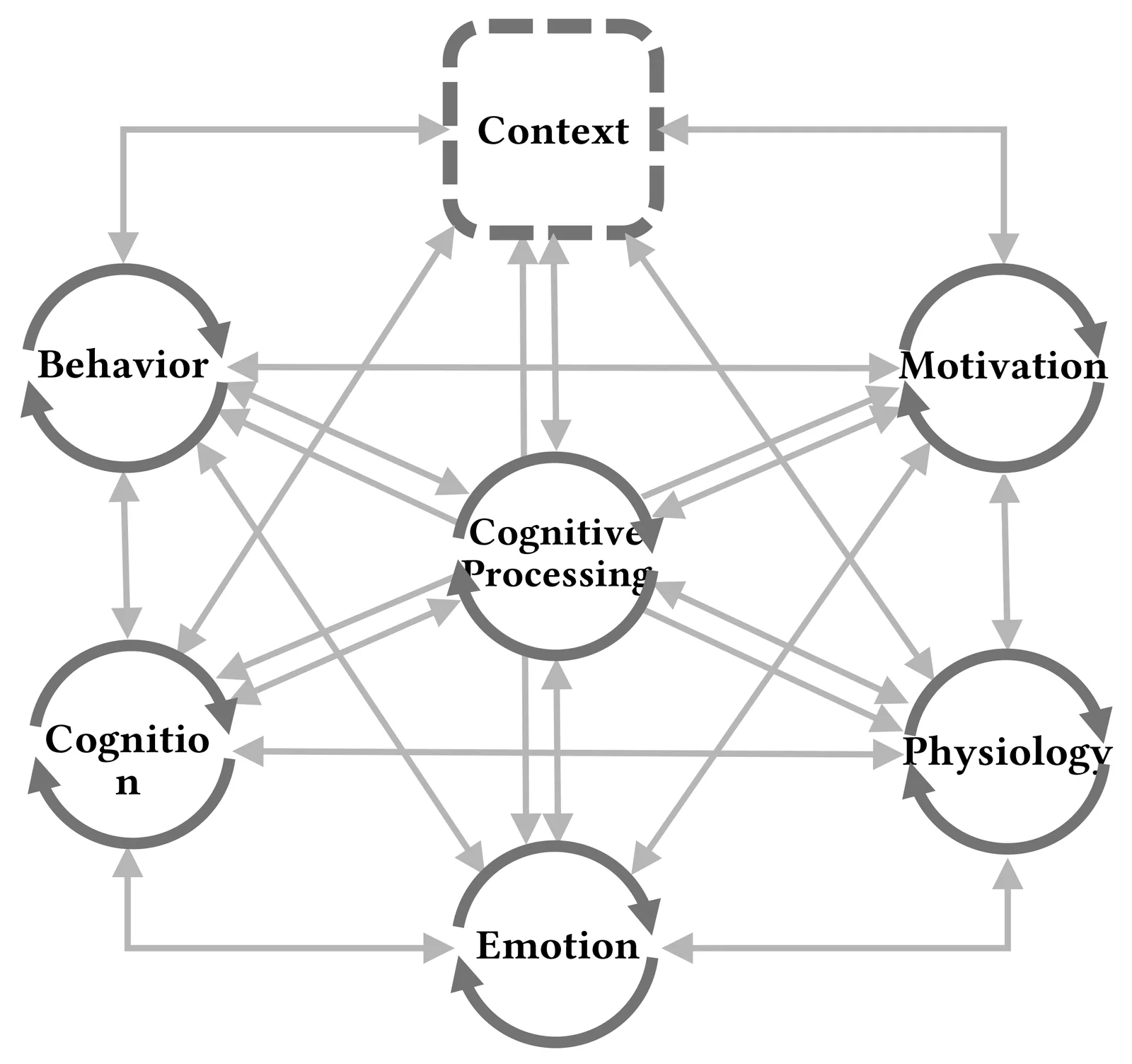
The Adaptive Process-Based Network Model (APNM)

As an alternative, the dynamic network approach advocates an idiographic view of psychological processes as interacting nodes of an individual network (Borsboom, 2017). While the dynamic network emphasizes causal relations and feedback loops within psychological systems, where certain nodes/symptoms activate others, the Extended Evolutionary Meta Model (Hayes et al., 2020) highlights the adaptiveness of human behavior and psychological functioning. An integration of both theories is provided by the Adaptive Process-Based Network Model (APNM), which postulates that adaptation results from the self-regulation and interaction of psychological processes conceptualized as nodes of an individual network. These processes can be described on the six core dimensions of cognition, emotion, physiology, behavior, cognitive processing, and motivation. The dimensions can be assessed in relation to a specific context using questions such as:

“How did I feel?” (e.g., sad vs. happy), “How was my body sensation?” (e.g., restless vs. calm), “How were my thoughts?” (e.g., „My life is hopeless.“ vs. „I can influence my life in a direction that I feel good about.”), “How was my behavior?” (e.g., approaching vs. avoidance), “How was my inner cognitive processing?” (e.g., ruminating vs. attentive), and “What motivational goal did I have in the situation?” (e.g., avoidance of failure vs. striving for appreciation). The resulting psychological networks may be adaptive or maladaptive depending on the context (see Figure 1).

In emotional disorders, maladaptation to the environment is also influenced by distorted cognitive processing. For instance, memory and attentional biases play a key role in the development and maintenance of emotional disorders (Cisler & Koster, 2010; Everaert et al., 2020; Phelps & Hofmann, 2019).

Although the reliability of measures used to assess cognitive processing in clinical practice remains questionable (Rodebaugh et al., 2016), treatment approaches targeting cognitive processes have been suggested to enhance therapeutic outcomes (e.g., Ehlers et al., 2012; Hvenegaard et al., 2020; Schreiber et al., 2015). In addition, research in neurocognitive science provides strong evidence for the substantial impact of motivational mechanisms on cognitive processing, emotions, and behavior (Braver et al., 2014). For instance, reward motivation has a robust influence on improving proactive cognitive control of goal-oriented behavior (Chiew & Braver, 2014). Furthermore, neurocognitive research has shown that motivational processes such as reduced reward responsiveness, via neural circuits, profoundly affect behavior and mental health problems like depression (Nestler, 2015).

Whereas psychological treatments have traditionally focused on maladaptive, psychopathological processes, defining “positive” and adaptive dimensions has become increasingly important in establishing treatment goals (Epton et al., 2017).

Thus, within a network approach to case conceptualization, it may be beneficial to specify not only maladaptive, but also adaptive poles of the dimensions relevant to the individual patient. Therefore, a dynamic network model may facilitate patient engagement in change processes, enhancing motivation to adopt the desired psychological processes and behaviors (Locke & Latham, 2002). So far, however, this approach has not yet been applied in the context of EMA. When applying continuous rating scales in EMA, however, it is important to consider potential psychometric issues such as floor effects.

In the evaluation of self-report measures, floor effects can limit sensitivity to detect variation at the lower end of the scale. A floor effect occurs when a substantial proportion of responses cluster at the lower bound, suggesting that the scale may not adequately capture lower levels of the construct of interest (de Vet et al., 2011; Terwee et al., 2007). This issue is particularly relevant in EMA contexts, where continuous scales (e.g., 0–100) are commonly used, and dynamic variation is critical for model interpretability. A floor effect is typically considered relevant when responses fall at or near the minimum score. For this study, we defined the floor range as the lowest 10% of the scale (i.e., values ≤ 10), allowing us to quantify and compare the prevalence of floor effects across experimental groups. This operationalization balances empirical convention with the specific characteristics of EMA data and continuous response formats.

In the present study, we investigated the feasibility of assessing maladaptive and adaptive psychological processes in an analogue sample of university students. We hypothesized that the inclusion of adaptive processes can be successfully implemented in EMA data used to calculate dynamic network models. Specifically, this approach is expected to provide a more holistic view of an individual’s experiences and associated processes compared to models based solely on maladaptive processes. A recent study by Nemani et al. (2025) provides evidence that the choice of bipolar versus unipolar scales in EMA significantly impacts the structural complexity of network models. The findings indicate that bipolar scaling captures a wider range of emotional and cognitive states, as participants are less likely to choose a neutral “0” response. This allows for a more detailed and accurate picture of psychological processes.

Previous research on emotion regulation and its impact on emotional experience suggests that the simultaneous assessment of both adaptive and maladaptive dimensions using EMA can reveal more nuanced patterns (De la Barrera et al., 2024; Short et al., 2018).

Building on these results, the present study examines how overall the variability in response distributions affects the density and connectivity of psychological networks. We hypothesize that network density will be greater when both maladaptive and adaptive processes are assessed, compared to when only maladaptive processes are considered.

Given that motivational schemas – as described above – control behavior and information processing, we hypothesize that motivational processes - ranging along a continuum from approach to avoidance – play a central role in understanding both maladaptive and adaptive psychological functioning and are characterized by a high degree of influence on other variables which is manifested by strong outgoing edges to other nodes in a dynamic network model. Furthermore, we propose that cognitive processing, due to its well-documented associations with a variety of psychological variables, represents another crucial node in dynamic networks of interconnected psychological processes, as indicated by strong associations with the other variables.

## Method

### Participants

Of the 50 individuals initially recruited, five participants discontinued the study before or during the EMA phase. The dropout reasons were not systematically recorded. The final sample consisted of 45 students from German universities who volunteered to participate in exchange for a small monetary compensation and the opportunity to gain experience with smartphone-assisted self-monitoring. Recruitment occurred via social media and digital flyers. By providing informed consent, participants confirmed that they were currently enrolled as university students and had access to a smartphone suitable for completing daily assessments. Participants ranged in age from 21 to 40 years (*M* = 25.19, *SD* = 4.65). The majority of the sample identified as female (73.6%), and over half (55.3%) were enrolled in psychology programs.

### Procedures

Participants received an email with a QR code to set up the EMA application and a questionnaire to identify dimensions of a personally relevant problem experienced in daily life. Participants collected data between October 2023 and January 2024 using the Vacay Status-PBT app [Mobile app] for iOS and Android (Vacay GmbH, 2020).

Participants were instructed to complete three daily assessments, each lasting about two minutes. They received at fixed times three prompts per day – 6 am, 12 pm, 6 pm. Each survey consisted of seven questions, with each question relating to one of the seven domains: emotion, cognition, situation, cognitive processing, motivation, bodily response, and behavior.

**Figure 2:**
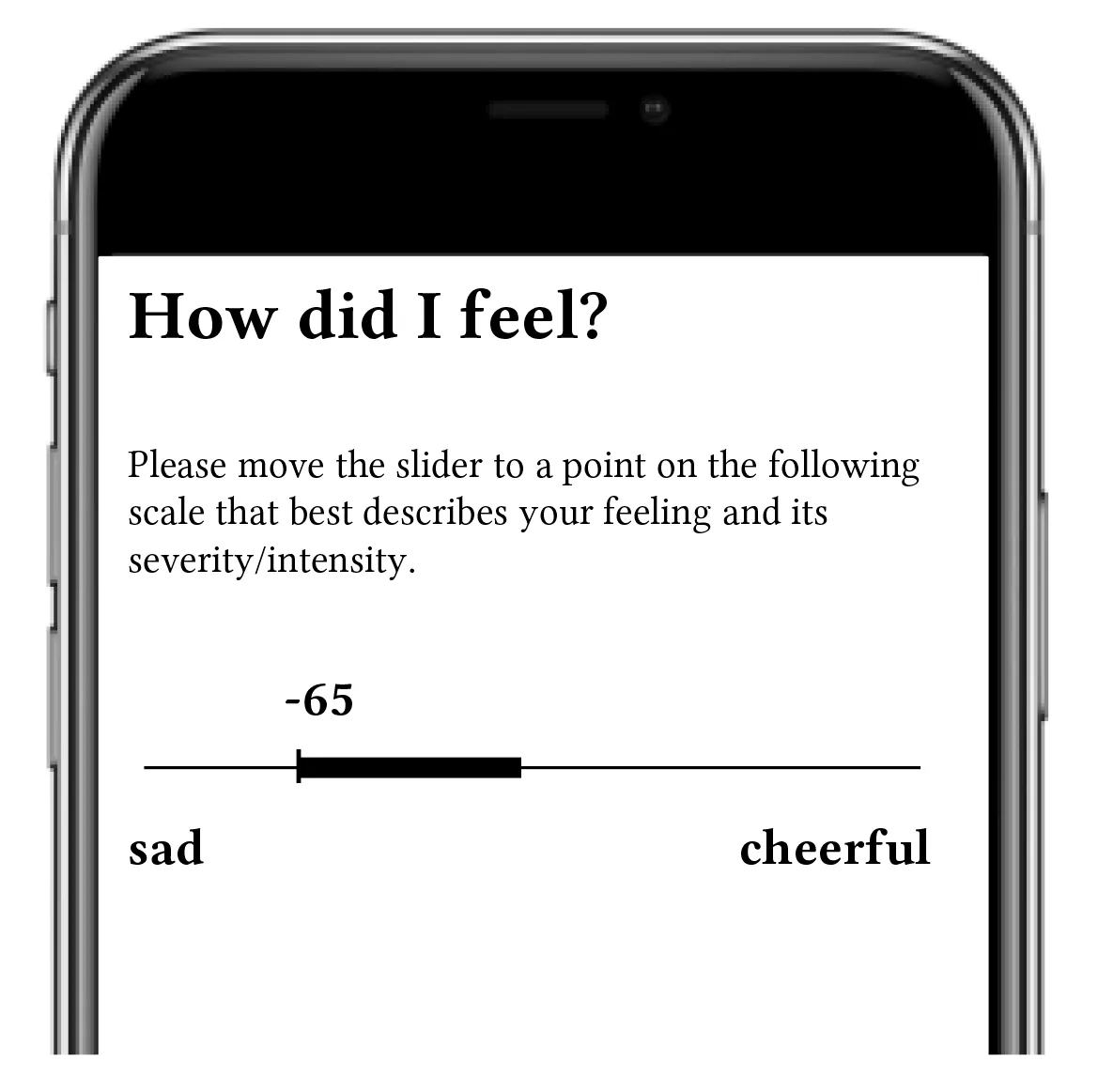
Example of an Individualized Item Assessing the Adaptive and Maladaptive Dimension of Emotion

Participants were randomly assigned to one of two conditions: one group (*n* = 23) specified maladaptive aspects and processes related to their identified problem, while the other group (*n* = 22) also specified corresponding adaptive, desirable processes. For the group that assessed maladaptive and adaptive dimensions, each question pertained solely to the specific process under investigation. For example: “How did I feel?” or “How was my body sensation?”. The items were arranged with two opposing poles placed at either end and a movable slider in between. The left pole represented the maladaptive expression of the process, while the right pole represented its adaptive form. As the slider was adjusted, numerical values appeared: moving it toward the maladaptive pole produced values from 0 to –100, while moving it toward the adaptive pole produced values from 0 to +100. Figure 2 shows how this was displayed in the app.

**Figure 3:**
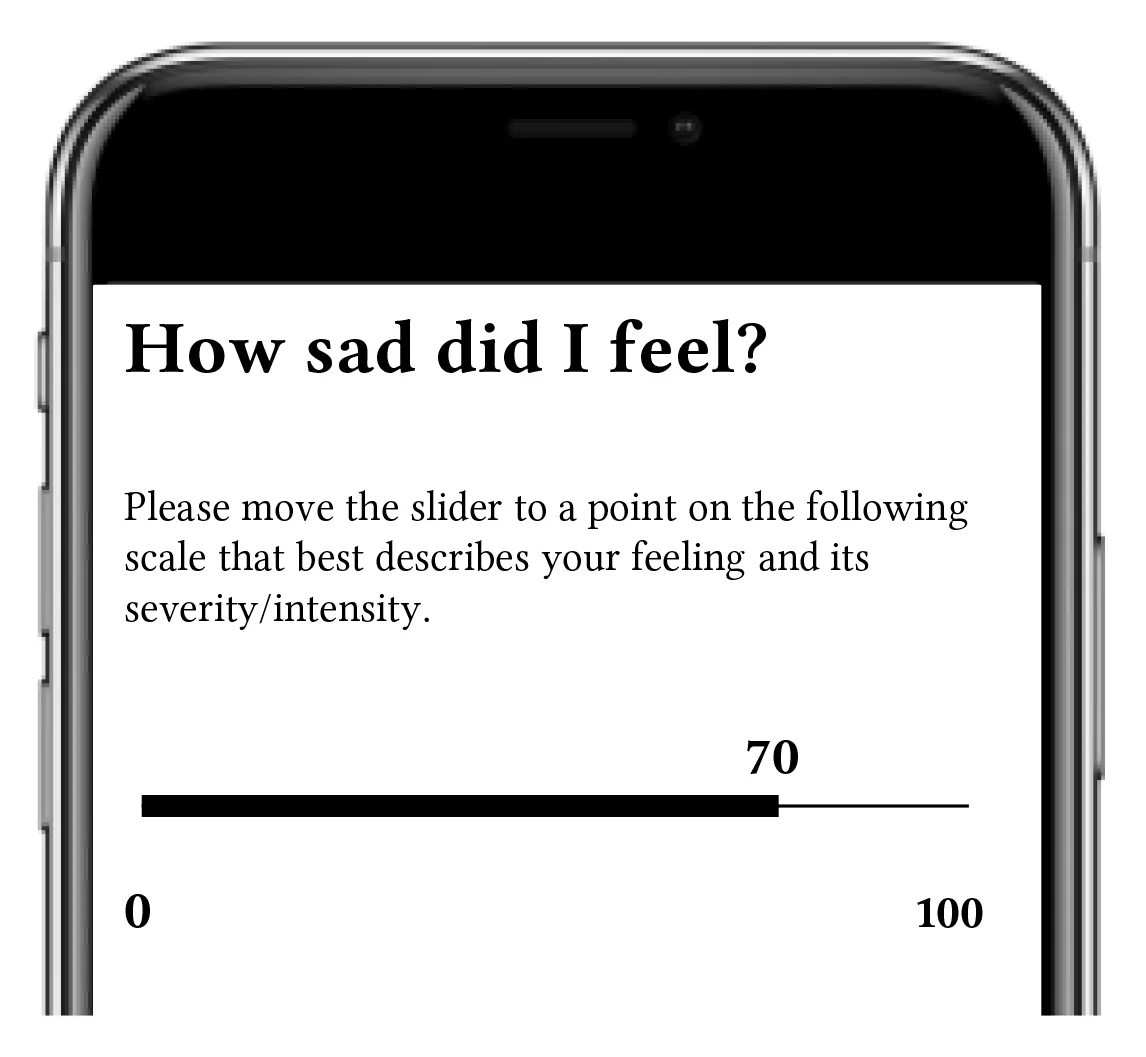
Example of the Individualized Item Assessing Only the Maladaptive Dimension of Emotion

For the group that assessed maladaptive dimensions only, questions contained the participant’s self-defined expression of the respective process. For example, if the individually defined feeling was “*sad*,” the corresponding question was: “*How sad did I feel?*” (Figure 3).

Participants were given substantial flexibility in the selection of anchors. For clarification and support in finding suitable descriptions for individual processes, they were given a questionnaire with examples of each domain available in Supplementary Material A.

Based on questionnaire responses, the research team programmed individualized EMA items in the app. Participants then began the EMA phase and were informed that they could request modifications to any item they felt did not accurately reflect their experience. This implied rewording items but also replacing predefined items with more appropriate ones. However, the project team took care that semantic-logical opposites of the condition-specific aspects were chosen, while also accounting for the subjectively perceived significance in the linguistic formulation. In cases where participants experienced difficulties, the issues were addressed through telephone consultations. The daily EMA protocol began with a situational anchoring item: “*How similar was the current, objective situation to my problem situation?*” followed by brief prompts assessing the seven identified dimensions (see Appendix A1 for the maladaptive group and Appendix A2 for the maladaptive-adaptive group), each rated using a visual analog scale. The length of the EMA phase was approximately five weeks on average but varied between participants and depended on the number of missings. In the maladaptive group, they participated on average for 42 days (*SD* = 8.74) and missed 18% of the beeps. In the maladaptive-adaptive group, they took part on average for 41 days (*SD* = 5.19) and missed 17% of the beeps. During the EMA phase, participants completed around 100 assessments (on average, 94 assessments in the maladaptive group and, depending on the variable 98/99 in the maladaptive-adaptive group) – aligning with prior research recommendations for idiographic modeling (Epskamp et al., 2018). During the EMA phase, the study team monitored participant entries. However, as monitoring was not performed daily, minor variations in the total number of measurement points occurred. Participants did not receive feedback and had no access to self-monitoring features.

The final evaluation yielded a total of 4363 EMA observations across all participants.

### Data Analysis

In order to evaluate the added value of including adaptive processes on a temporal group level, multilevel vector autoregressive (mlVAR) models were calculated. For the purpose of this study, we focused on the fixed-effect temporal networks (using a lag-1 approach) to model the average temporal relationships within individuals. The associations between variables (“nodes”) will be referred to as “edges”, with a blue edge indicating a positive association and a red edge a negative association. As a measure of relative importance, outStrength refers to the sum of the weights of all outgoing edges from a specific node. InStrength refers to the sum of the weights of all incoming edges to a specific node, representing how strongly that node is influenced by other nodes.

The main data analysis was conducted using the mlVAR package (Version 0.5.2; Epskamp et al., 2024) within RStudio (Version 2024.04.2, Posit Team). Prior to analysis, important preprocessing steps included the removal of the linear time trends through detrending and the handling of missing data using the Kalman filter (function na_kalman) from the imputeTS package (Version 3.3; Moritz & Bartz-Beielstein, 2017). For each participant and each variable, missing data were below 40%.

## Results

### Descriptive Statistics

Descriptive statistics for both groups are summarized in Table 1. The maladaptive group showed mean scores ranging from 38.64 (Behaviour) to 49.17 (Motivation), with standard deviations around 29 to 32, indicating moderate within-group variability. The maladaptive-adaptive group exhibited scores centered closer to zero across most domains, with notably higher variability (*SD* mostly > 50), reflecting a more diverse range of responses. The broader scale range (-100 to +100) for the maladaptive-adaptive group compared to the restricted positive range (zero to 100) for the maladaptive group further highlights the distinct response patterns between groups. Intraclass correlation coefficients (ICCs) calculated quantified the proportion of variance attributable to stable between-person differences across psychological domains (Siepe et al., 2025). The maladaptive group showed a mean ICC of .389 (*SD* = 0.05), with values ranging from .319 (Behaviour) to 0.465 (Body Response). The maladaptive-adaptive group demonstrated a slightly lower mean ICC of .340 (*SD* = 0.05), with the highest stability observed for Situation (.445) and lowest for Cognitive Processing (.289). These moderate ICC values indicate that approximately 34 to 39% of total variance stems from consistent individual differences, while the majority (666%) reflects within-person fluctuation.

**Table 1:** Summary of Descriptive Statistics for Items by Group

Group	Item	*M*	*SD*	*Mdn*	Min	Max	*N*
1	Behaviour	38.64	31.34	31.82	0	100	2069
1	Body Response	45.65	31.80	45.04	0	100	2068
1	Cognition	40.57	30.01	35.00	0	100	2069
1	Emotion	40.77	29.26	35.00	0	100	2068
1	Motivation	49.17	32.45	57.00	0	100	2068
1	Cognitive Processing	45.98	30.40	45.00	0	100	2069
1	Situation	49.13	30.78	57.00	0	100	2071
2	Behaviour	-0.36	53.00	0.00	-100	100	2287
2	Body Response	0.35	55.80	-6.20	-100	100	2286
2	Cognition	-5.45	54.27	-15.45	-100	100	2288
2	Emotion	-1.34	51.95	-9.70	-100	100	2288
2	Motivation	-1.02	54.92	-6.60	-100	100	2283
2	Cognitive Processing	0.54	56.14	0.00	-100	100	2287
2	Situation	45.04	36.99	50.00	-100	100	2246

*Note*. Group 1 = Maladaptive (*n* = 23) with raw scores (zero to 100); Group 2 = Maladaptive Adaptive (*n* = 22) with standardized scores (-100 to 100). *SD* = Standard Deviation; *N* = total number of participants. Chi-square tests were used to compare groups.

To examine potential multicollinearity and node redundancy, zero-order correlation matrices among the seven key domains were calculated for each group. In the maladaptive group, correlations ranged from *r* = .33 to *r* = .74, indicating moderate associations but also sufficient distinction among nodes. For the maladaptive-adaptive group, correlations spanned from approximately *r* = -.54 to *r* = .81.

### Network Analysis of EMA Variables

The data was analyzed separately for each group: a group of participants which exclusively recorded maladaptive processes and the other group that assessed maladaptive and adaptive processes. Between-person networks, reflecting trait-like associations among participant averages, are presented in Supplementary Material B in Figure 1 and Figure 2.

#### Fixed Effects Temporal Networks (Maladaptive/Maladaptive-Adaptive)

**Figure 4:**
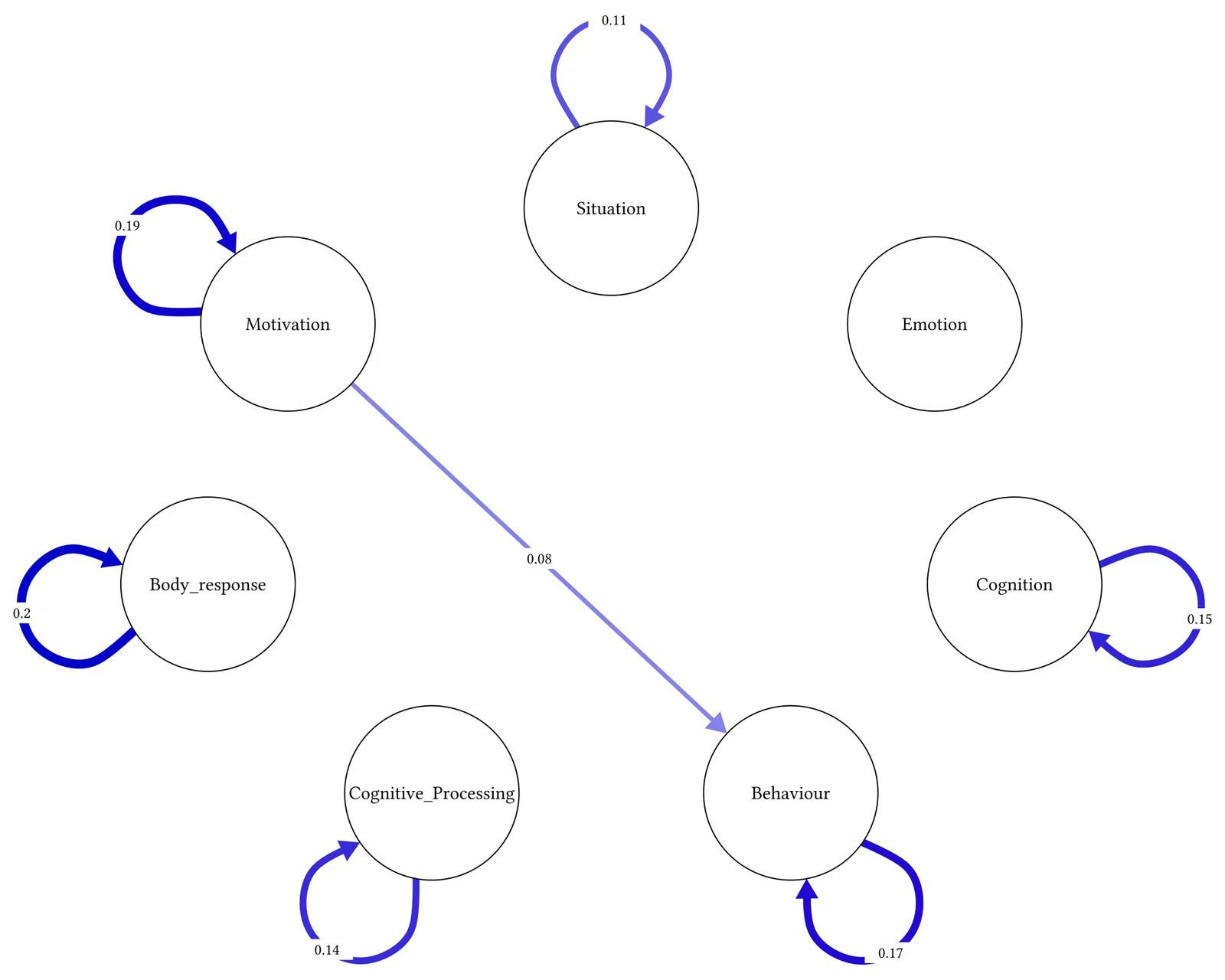
Fixed Effects Temporal Network, Maladaptive Processes *Note.* Thickness of the autoregressive loops indicates self-influence strength. Thickness of the arrow indicates association strength. Arrowheads indicate the direction of the association between nodes (outStrength or inStrength). Arrow colour indicates whether the association is positive (blue) or negative (red). Higher colour saturation indicates a stronger association.

**Figure 5:**
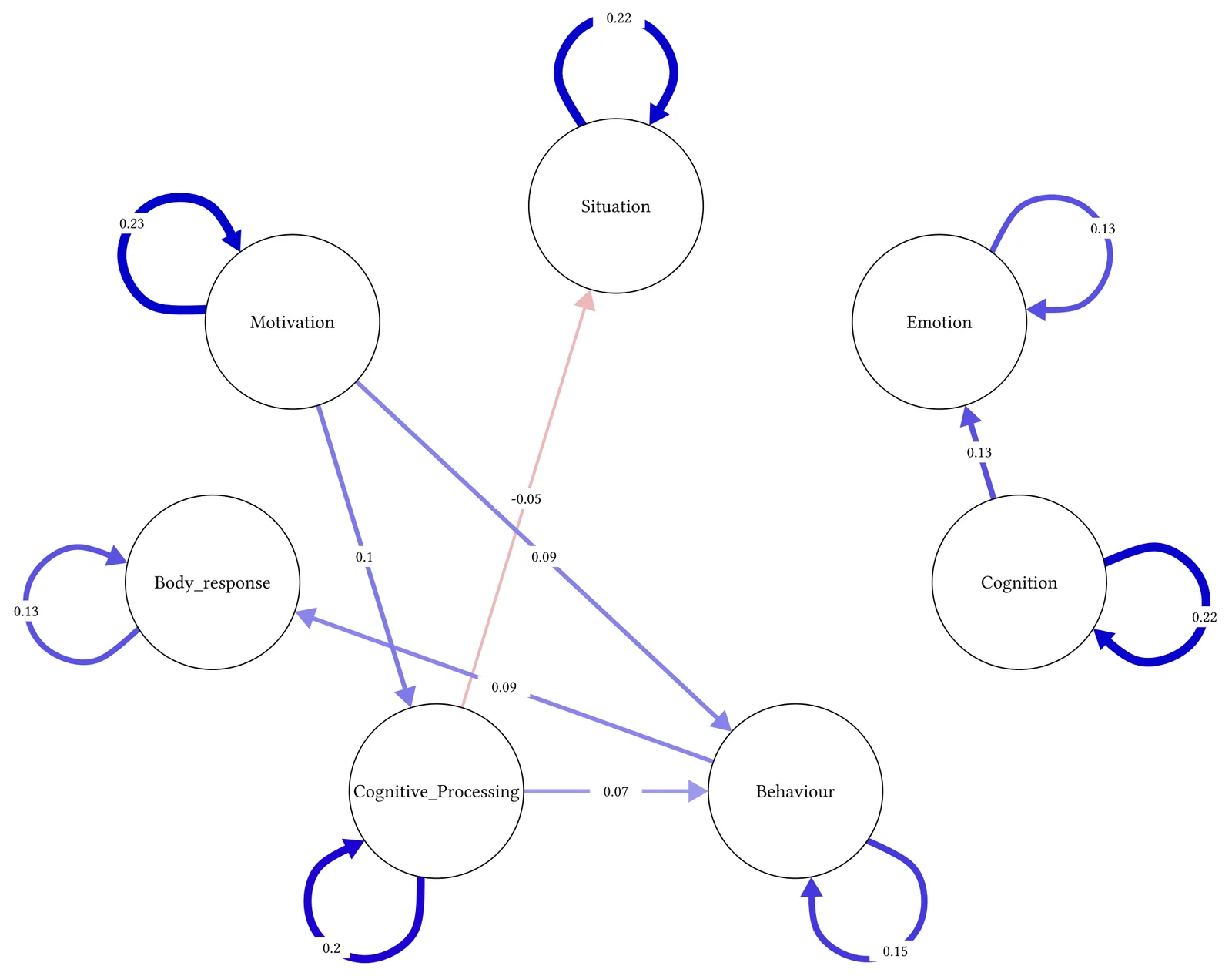
Fixed Effects Temporal Network, Maladaptive – Adaptive Processes *Note*. Thickness of the autoregressive loops indicates self-influence strength. Thickness of the arrow indicates association strength. Arrowheads indicate the direction of the association between nodes (outStrength or inStrength). Arrow colour indicates whether the association is positive (blue) or negative (red). Higher colour saturation indicates a stronger association.

Figures 4 and 5 show the results of the two fixed effects temporal networks – Figure 4 for the group with exclusively maladaptive networks and Figure 5 for the group with maladaptive and adaptive processes.

#### Density

In the fixed-effect temporal maladaptive network, 14% of all possible edges were present, compared to 27% in the adaptive-maladaptive network. While some nodes in the average maladaptive network remained isolated, all nodes in the adaptive network were interconnected.

In Figure 4, which depicts the average temporal network of the maladaptive group, the *Emotion* node exhibits no temporal associations with any other variables. Most of the remaining nodes display only auto-temporal associations. Notably, the only cross-variable temporal link is a positive association from *Motivation* to *Behavior*, suggesting that, on average, motivation temporally predicts subsequent behavior across measurement points.

In Figure 5, which presents the average temporal network of the maladaptive-adaptive group, two interconnected subsystems emerge. In the first, *Cognition* is positively associated with *Emotion*. In the second, *Motivation* appears to initiate adaptive cascades, showing positive temporal associations with both *Behavior* and *Cognitive Processing*. *Cognitive Processing*, in turn, is negatively linked to *Situation* and positively associated with *Behavior*, which then shows a positive temporal connection to *Body Response*. Additionally, all nodes in the network display autoregressive loops. These reflect that each construct’s state at one time point significantly predicts its own state at the next, demonstrating temporal consistency within that psychological dimension. Notably, in an idiographic network model with bipolar scaling, the network structure alone does not reveal which variable manifestations influence each other over time. To determine this, the individual values must be examined.

The estimation of the fixed effects temporal networks showed *Motivation* had the highest outStrength in both groups, which agrees with our hypothesis that motivation strongly impacts other psychological processes and should be targeted. The highest inStrength is in *Behavior* (see Table 2).

**Table 2:** OutStrength and inStrength of the Fixed Effects Temporal Network for Both Groups (Maladaptive; Maladaptive-Adaptive)

Variable	Maladaptive (*n* = 22)		Maladaptive – Adaptive (*n* = 23)	
	outStrength total	inStrength total	outStrength total	inStrength total
Situation	0.110	0.110	0.222	0.273
Emotion	0.000	0.000	0.134	0.267
Cognition	0.151	0.151	0.351	0.218
Behaviour	0.165	0.247	0.245	0.321
Cognitive Processing	0.145	0.145	0.323	0.297
Body Response	0.200	0.200	0.130	0.222
Motivation	0.266	0.185	0.423	0.230

*Note.* OutStrength = sum of the weights of all outgoing edges from a node; InStrength = sum of the weights of all incoming edges to a node. Higher values indicate stronger connections.

### Response Distribution and Its Impact on the Network Structure

**Figure 6:**
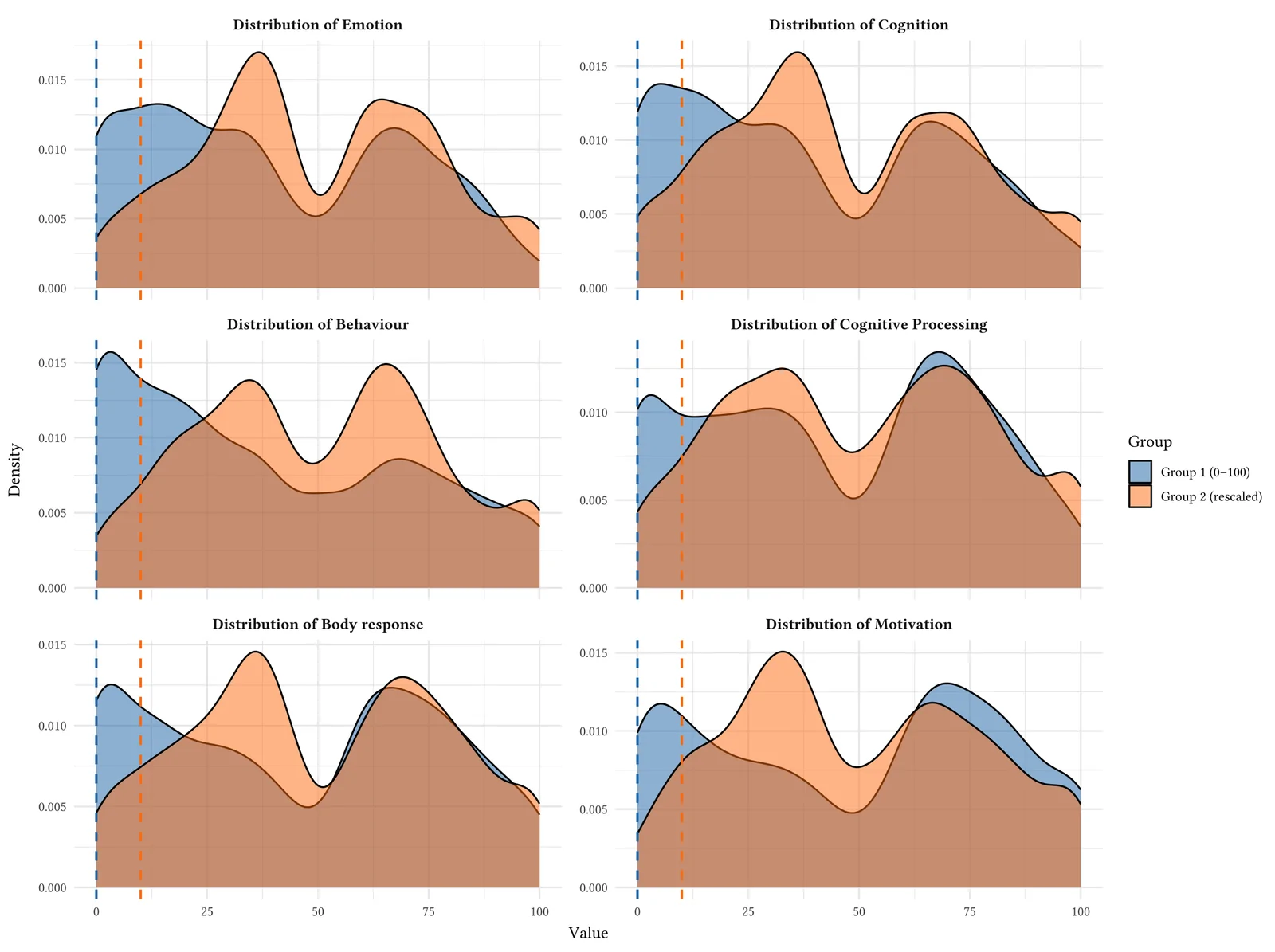
Distribution of Data in the Two Groups Maladaptive and Adaptive-Maladaptive *Note.* Group 1 = maladaptive-only group; Group 2 = maladaptive–adaptive group. Values in Group 2 were linearly transformed from −100–0, 0–50, and 100–100 to a unipolar 0–100 scale to match the maladaptive-only group. The dotted lines indicate the floor range (lowest 10% of the scale).

To ensure comparability across groups and to examine potential differences in floor effects, all bipolar scales ranging from –100 to +100 (used in the maladaptive-adaptive group) were linearly rescaled to match the unipolar 0 to 100 formats employed in the maladaptive only group. The transformation was linear, mapping –100 to zero, zero to 50, and +100 to 100. Thus, both groups were expressed on the same 0 to 100 metrics, despite the original difference in raw scale range. This transformation allowed for a uniform definition of the lower 10% of the scale (≤ 10) as the operational threshold for floor effects across all variables (Figure 6). We then analyzed response distributions across all scales to assess whether more evenly distributed responses across the scale increased network density. Results revealed that participants in the adaptive-maladaptive group exhibited a significantly more balanced response distribution compared to the maladaptive-only group (*p* < .05) suggesting that the inclusion of adaptive processes encourages a broader range of responses across the scale, rather than clustering at extreme values.

Individuals with greater response dispersion exhibited higher network connectivity, indicating that broader variability enables more nuanced psychological interconnections. By contrast, those with narrow response distributions (i.e., excessive clustering in specific scale regions) demonstrated lower network density, reinforcing the notion that response flexibility enhances the structural integrity of network models. Adaptive–maladaptive networks consistently exhibited significantly fewer floor responses across all variables compared to maladaptive-only networks (Table 3). This indicates enhanced sensitivity and greater response variability when adaptive processes are included in EMA assessments. Chi-square tests revealed highly significant differences between groups (all *p* < .001).

**Table 3:** Comparison of Floor Effects of the Maladaptive (n = 2,070) and Maladaptive-Adaptive (n = 2,287) Group

Variable	Maladaptive		Maladaptive-Adaptive		χ^2^	*p*
	Floor count	Floor percent	Floor count	Floor percent		
Behavior	513	24.8%	145	6.34%	287.05	< .001
Body Response	407	19.7%	186	8.14%	122.02	< .001
Cognition	435	21.0%	179	7.82%	155.31	< .001
Emotion	402	19.4%	151	6.60%	160.40	< .001
Motivation	379	18.3%	155	6.79%	133.09	< .001
Cognitive Processing	348	16.8%	175	7.65%	85.55	< .001

*Note*. Chi-square tests were used to compare groups.

## Discussion

Building on previous studies on EMA (Mink et al., 2025; Nemani et al., 2025; Yim et al., 2020), the present study demonstrates the feasibility of incorporating both maladaptive and adaptive processes into the collection of EMA data for the calculation of idiographic dynamic network models. The present study advances existing research on EMA as a methodological approach to data collection and provides a foundation for subsequent investigations in clinical populations. Future work could specifically evaluate its applicability and utility for individualized case conceptualization as the implementation in psychotherapeutic settings is theoretically useful but still lacking (Mink et al., 2025).

The findings provide evidence that including adaptive processes enhances the richness of EMA data, used in network analysis. For example, when assessing emotion exclusively on maladaptive dimensions, emotional states lacked temporal stability and associations with other psychological processes, indicating that maladaptive emotion alone may not sufficiently capture the dynamic interplay within individuals. Furthermore, increased richness of EMA data is reflected by greater response dispersion and increased connectivity between variables in the dynamic network model. This in turn contributes to a deeper understanding of interrelationships among psychological processes and supports our hypothesis that density is stronger when both maladaptive and adaptive processes are assed.

It should be noted that in the present study, network density is operationalized as the average absolute strength of temporal (lagged) connections and thus primarily reflects the magnitude of direct interdependencies rather than network topology or functional optimality. When networks predominantly capture maladaptive processes, higher density may indicate increased vulnerability, as strongly coupled systems can be more sensitive to external perturbations and show reduced flexibility (Borsboom, 2017). However, in the present context, increased density is primarily interpreted as reflecting enhanced response variability and reduced floor effects due to the inclusion of adaptive dimensions, which facilitates the detection and interpretation of temporal associations. Because density is based on absolute edge weights, it does not distinguish between stabilizing and destabilizing dynamics, underscoring the need for cautious interpretation (Fried & Cramer, 2017).

By incorporating adaptive processes, the narrow definition of well-being as merely the absence of suffering is challenged, allowing for a more holistic understanding of an individual's experiences (Joseph & Wood, 2010). This aligns with the growing acceptance of a dimensional view on psychological processes and the idea that the severity of psychopathology varies along a continuous spectrum (Clark et al., 2017; van Os et al., 2009). Practically, adding adaptive processes in EMA, broadens the perspective on processes relevant to everyday life. In our study, motivation emerged as the central node in both groups, supporting our hypothesis of its high impact on other variables in a dynamic network model.

Thus, assessing approach motivation seems to be particularly important, as it may enhance intrinsic motivation, which mediates links between clinical symptoms and social functioning (Yamada et al., 2010) and drives behavior aimed at fulfilling psychological needs (Ryan & Deci, 2000).

Memory, attention, and consciousness have been proposed as crucial components of cognitive processing that significantly influence psychological functioning and mental health (Becker et al., 2021; Knowles et al., 2016; Phelps & Hofmann, 2019). Several dimensions of cognitive processing, such as concrete versus abstract thinking modes and rumination (Watkins & Moulds, 2005), as well as emotion regulation strategies like attentional deployment (McRae & Gross, 2020), have been linked to the development of emotional disorders. In contrast, cognitive processing styles such as decentering and mindful attention (Bernstein et al., 2015) have been proposed as adaptive responses to distress and are considered key targets in various psychological treatment approaches, including the Third Wave of CBT. Given that cognitive processes are fundamental to the mechanisms maintaining psychological problems and symptoms, it has been proposed that personalized clinical case formulation should address not only dysfunctional cognitive content (e.g., beliefs and automatic thoughts), but also dysfunctional cognitive processes (e.g. attentional biases; Dennis-Tiwary et al., 2019). However, due to their implicit nature, the identification of memory and attentional processes often requires substantial support, such as guidance from a therapist and intensive self-monitoring to recognize automatic patterns (Stefana et al., 2024). Therefore, the results of the present study support efforts to incorporate cognitive processing as a potential target for enhancing adaptive functioning within ecological momentary assessment frameworks by showing that cognitive processing has connections to other variables in a dynamic network model. In the present study, the connections are not particularly strong compared to others. Further research is needed to explore potential underlying mechanisms. Incorporating measures such as reflective functioning (Fonagy et al., 2016) could reveal whether interindividual differences in data quality are moderated by the capacity to reflect on internal states (Fonagy et al., 2016). In general, we recommend considering motivational processes as well as cognitive processing when designing future research.

For decades, different therapy schools used different approaches to case conceptualization. While in psychodynamic therapy the assessment of central psychodynamic constructs is crucial (Luborsky et al., 1994), the SORCK model (Kanfer & Saslow, 1965) is an established model for case conceptualization in behavior therapy. The transdiagnostic approach is gaining increasing popularity, because it pushes traditional diagnostic boundaries and offers a deeper understanding of the structure of psychological problems (Dalgleish et al., 2020). The findings of our study, which support a more in-depth exploration using bipolar scales, align with the broader shift to a more comprehensive understanding of interacting psychological processes. This approach can serve as a fundament for case conceptualization in the context of personalized psychotherapy, an area of growing importance (Hayes et al., 2020; Huibers et al., 2021).

Nemani et al. (2025) demonstrated that bipolar scaling reduces zero inflation, our findings suggest that overall response variability across the entire scale plays a critical role in determining network connectivity. Specifically, the concentration of responses in extreme boundary regions (e.g., zero to 10, 90 to 100, -10 to 0, -100 to -90) may constrain network formation, limiting meaningful interconnections between psychological variables.

Our findings extend previous research by demonstrating that the additional assessment of adaptive processes can reduce floor effects which are associated with restrictions in the possibility to capture fluctuations in symptom ratings (Mestdagh et al., 2018) and limited variance (Šimkovic & Träuble, 2019). Since values at the lower bound cannot decrease further (e.g., Fries et al., 2014; Šimkovic & Träuble, 2019), floor effects constrain the ability to detect longitudinal changes in the lower range of the scale. By incorporating adaptive processes into the scale, overall variability is increased, thereby reducing constraints on the detection of interconnections among psychological variables. Our findings suggest examining both adaptive and maladaptive dimensions to gain a comprehensive understanding of the interplay among psychological processes.

This study has several limitations that should be acknowledged. First of all, temporal associations and node centrality metrics derived from network analyses represent statistical dependencies rather than causal effects. Therefore, conclusions about mechanisms should be made with caution. Network analysis conceptualizes psychological phenomena as systems of interacting symptoms or variables rather than manifestations of a single latent construct (Bastiaansen et al., 2020). One the one hand, this approach allows researchers to explore direct relationships among variables, identify central nodes, and detect possible pathways of change, offering insights for both theory and intervention (Bringmann et al., 2019). On the other hand, the method faces important limitations, particularly regarding the stability and replicability of estimated networks, which may vary with sample size, measurement error, and analytic choices (Hevey, 2018). As the robustness of the estimated networks was not systematically evaluated, the interpretation of the findings should pay attention to this fact.

In addition, adaptive and maladaptive processes were assessed in relation to the specific context in which the problem typically arises. This approach aligns with research showing coping effectiveness depends strongly on context (Cheng, 2001; Cole et al., 1994; Gross, 1998, Kalokerinos et al., 2017). However, this context-specific focus also limits the generalizability of our findings. Bonanno (2021) argues that psychological flexibility – the ability to select the most appropriate coping strategy based on situational demands – is a key factor in successfully coping with stressful events. This perspective may be especially relevant for EMA, where focusing on flexibility over rigid maladaptive–adaptive distinctions could offer more nuanced insights.

Furthermore, the sample primarily consisted of female university students, resulting in both a gender imbalance and limited demographic diversity.

Additionally, the relatively small sample size (*N* = 45), although comparable to feasibility samples in EMA research, may limit statistical power to detect subtle group differences in multilevel vector autoregressive modeling. Future research should aim to replicate these findings in larger samples, with a more balanced gender distribution and diverse educational backgrounds.

Another limitation concerns the method of participant recruitment: individuals volunteered to participate and were given the opportunity to engage in smartphone-based self observation which may have introduced a bias, as participants were likely already interested in using ecological momentary assessment (EMA) tools which may not represent clinical populations.

Furthermore, an important limitation of the study is the reliance on self-report measures, which may be influenced by social desirability or lack of awareness.

Additionally, given the relatively young and tech-savvy nature of the sample, the ease of smartphone use observed in this study may not be representative of other populations, potentially limiting the applicability of these findings to broader groups. A further limitation is the lack of procedures to identify or address careless responding in EMA, which may add noise and reduce the accuracy and interpretability of the dynamic network models.

Another limitation is the uncertainty associated with centrality estimates derived from mlVAR models. The complexity of multilevel temporal modeling combined with our relatively small sample size and the variability inherent in EMA data contribute to potential imprecision. As a result, while motivation appeared as a central node, the robustness of this finding remains uncertain. Future research employing bootstrap-based stability analyses is needed to strengthen conclusions about key intervention targets.

Moreover, as our sample consisted of non-clinical university students, reduced floor effects may partly reflect lower baseline symptom severity and greater variability in adaptive functioning in this population. Replication in clinical samples is needed to determine whether including adaptive processes in EMA yields the same benefits under higher symptom burden and lower functioning.

Finally, the use of different scale formats: zero to 100 in the maladaptive group and –100 to +100 in the maladaptive–adaptive group is another limitation. Although responses were linearly rescaled for comparability, the interpretation of the anchors and the understanding of the midpoint of the scale, which may not consistently reflect a true “neutral” state, may vary. This interpretative variability could introduce noise or bias in the data, potentially affecting the observed differences between the two groups. Future studies should standardize formats to rule out this source of bias.

## Supplementary Materials

The Supplementary Materials contain the following items:

- **R code** used for the main analyses (Hufschmidt, 2026S)
- **Appendix** (Hufschmidt et al., 2026S): The appendix associated with this article provides the EMA-items used for assessing the maladaptive dimensions and the maladaptive and adaptive dimensions of individual psychological processes.
- **Additional information, tables, and figures** (Hufschmidt et al., 2026S): The supplementary material contains an additional information sheet provided to participants to help them define their own psychological processes (*Supplementary Material A*) and a comprehensive set of tables and figures that exceeded the scope of the main article (*Supplementary Material B*).

HufschmidtB.
 (2026S). Adaptive and maladaptive networks using ecological momentary assessment
[R code]. PsychOpen. https://osf.io/7amzu


HufschmidtB.
EbertM.
NemaniA.
KohlV.
PahlenL.
MüllerS.
HofmannS. G.
StangierU.
 (2026S). Supplementary materials to "Adaptive and maladaptive networks using ecological momentary assessment"
[Appendix; Additional information, tables, and figures]. PsychOpen. 10.23668/psycharchives.21827


## Data Availability

The data underlying the results presented in this study are available from the Department of Clinical Psychology and Psychotherapy at Goethe University Frankfurt (contact via stangier@psych.uni-frankfurt.de). The data cannot be shared publicly due to ethical restrictions and privacy concerns regarding participant confidentiality. Access to these data may be granted by the Goethe University Institutional Data Access/Ethics Committee for researchers who meet the criteria for access to confidential data. The R code used for the main analyses is available in OSF at https://osf.io/7amzu

## Preregistration

Due to the small size the study was not submitted to a preregistration platform.

## Reporting Guidelines

The study adheres to the JARS-Quant Guidelines of the American Psychological Association.

## Social Media Accounts

*Bettina Hufschmidt:* LinkedIn
