# Correction of Bettina Hufschmidt et al. (2026). Adaptive and Maladaptive Networks Using Ecological Momentary Assessment

**DOI:** 10.32872/cpe.23516

**Published:** 2026-08-31

In the published version of

HufschmidtB.
EbertM.
NemaniA.
KohlV.
PahlenL.
MüllerS.
HofmannS. G.
StangierU.

(2026). Adaptive and maladaptive networks using ecological momentary assessment.
Clinical Psychology in Europe,
8(2), e17891.
10.32872/cpe.17891

the statement on Competing Interests was incomplete. Therefore, the authors would like to replace the published Competing Interests statement with the following:

**Competing interests:** The authors declare that there is no conflict of interest regarding the publication of this article. None of the authors have any financial, personal, or professional relationships that could be perceived as influencing the research presented in this study. Dr. Hofmann receives financial support by the Alexander von Humboldt Foundation (as part of the Alexander von Humboldt Professur); the Hessische Ministerium für Wissenschaft und Kunst (as part of the LOEWE Spitzenprofessur); the DYNAMIC center, funded by the LOEWE program of the Hessian Ministry of Science and Arts (Grant Number: LOEWE1/16/519/03/09.001(0009)/98); the Deutsche Forschungsgemeinschaft (DFG, German Research Foundation) – Project-ID 521379614 – TRR 393/SFB Transregio 393; and the Excellence Cluster EXC3066 "The Adaptive Mind" (Project No. 533717223). He also receives compensation for his work as editor from the American Psychological Association and royalties and payments for his work from various publishers.

